# Immune Checkpoint Inhibitors for Vaccine Improvements: Current Status and New Approaches

**DOI:** 10.3390/pharmaceutics14081721

**Published:** 2022-08-17

**Authors:** Alexander Batista-Duharte, Fakhri Hassouneh, Pablo Alvarez-Heredia, Alejandra Pera, Rafael Solana

**Affiliations:** 1 GC01 Immunology and Allergy Group, Maimonides Biomedical Research Institute of Cordoba (IMIBIC), 14004 Cordoba, Spain; 2 Department of Cell Biology, Physiology and Immunology, University of Cordoba, 14004 Cordoba, Spain; 3 Immunology and Allergy Service, Reina Sofia University Hospital, 14004 Cordoba, Spain

**Keywords:** immune checkpoint inhibitors, molecular adjuvants, vaccines, monoclonal antibodies, antisense oligonucleotides, aptamers, peptides, CTLA-4, PD-1, PD-L1

## Abstract

In recent years, the use of immune checkpoint inhibitors (ICIs) in combination with approved or experimental vaccines has proven to be a promising approach to improve vaccine immunogenicity and efficacy. This strategy seeks to overcome the immunosuppressive mechanisms associated with the vaccine response, thereby achieving increased immunogenicity and efficacy. Most of the information on the use of ICIs combined with vaccines derives from studies on certain anti-tumor vaccines combined with monoclonal antibodies (mAbs) against either cytotoxic T lymphocyte-associated protein 4 (CTLA-4), programmed cell death protein 1 (PD-1), or programmed death-ligand 1 (PD-L1). However, over the past few years, emerging strategies to use new-generation ICIs as molecular adjuvants are paving the way for future advances in vaccine research. Here, we review the current state and future directions of the use of ICIs in experimental and clinical settings, including mAbs and alternative new approaches using antisense oligonucleotides (ASOs), small non-coding RNAs, aptamers, peptides, and other small molecules for improving vaccine efficacy. The scope of this review mainly includes the use of ICIs in therapeutic antitumor vaccines, although recent research on anti-infective vaccines will also be addressed.

## 1. Introduction

Vaccination is widely considered one of the greatest achievements of modern medicine. Vaccines save millions of lives and protect millions more from getting sick. Since ancient times, the use of immunization practices has shown its effectiveness in the prevention of infectious diseases. Due to the lack of knowledge of the protection mechanisms induced by those first formulations, those initial practices were empirical and not exempt from failures [1].

With the advancement of knowledge in immunology, it has been possible to obtain increasingly effective and safer vaccines. However, despite the significant progress achieved so far, more innovations are needed. Each year, roughly three million individuals still die of vaccine-preventable diseases, including respiratory infections, tuberculosis, diarrheal diseases, malaria, and others, especially in low-income countries [2]. Other challenges consist of obtaining antitumor vaccines, and more effective vaccines for older people [3].

One of the strategies to achieve effective vaccines has been the use of immunological adjuvants. An adjuvant is a substance that is added to a vaccine to stimulate and enhance the quality, magnitude, and durability of the specific immune response [4]. The first adjuvant used in human vaccines was aluminum hydroxide in 1919, and for seven decades, it was the only adjuvant approved for this purpose. Since the 1990s, only five more adjuvants have been included in licensed vaccines [5]. Although many other compounds have demonstrated high potency in preclinical studies, they have not been licensed to be used in humans, principally owing to safety concerns [6,7]. This means that a lot of resources and time have been invested in research that ultimately does not lead to products approved for clinical use. Even today the molecular mechanisms by which adjuvants that are approved for clinical use work remain only partially understood [4].

Currently, there is great interest in developing adjuvants with well-defined mechanisms of action, targeting known pathways involved in the immune response, which allows optimization of vaccine formulation designs and a better evaluation of pharmacological response and toxicity [8,9]. Molecular adjuvants are single and well-characterized molecules that function as regulators of the immune response through several mechanisms, including activation of the innate immune system, improvement of the antigen presentation by professional antigen-presenting cells (APCs), proliferation, differentiation, and maturation of innate immune cells and lymphocytes [10]. These substances exert their action by their interaction with signaling molecules such as cytokines, costimulatory molecules, toll-like receptors, inflammasome (and other innate immunity cytosolic surveillance pathways), or immune checkpoints. The greatest experience in modulating these immune control pathways has been based on the use of monoclonal antibodies. New approaches, including gene knockdown and epigenetic control, have also contributed to increasing the range of compounds as molecular adjuvants [11,12].

In recent years, the use of immune checkpoint inhibitors (ICIs) has become one of the most important strategies in cancer management and with it, there has been a significant change in the cancer treatment landscape [13]. The first-generation’ ICIs consist of monoclonal antibodies (mAbs) targeting the Cytotoxic T-Lymphocyte Antigen 4 (CTLA-4), the programmed cell death receptor-1 (PD-1), and the programmed cell death ligand-1 (PD-L1). These ICIs have revolutionized the treatment of a variety of types of cancer, with improved clinical outcomes compared to classic anti-tumor treatments [14]. However, therapeutic success is currently restricted to a limited number of patients [15]. Thus, diverse strategies are being optimized by applying suitable vaccine preparations combined with ICIs to achieve stronger immunogenicity and efficacy [16,17]. These combinations are under clinical investigation, and, in general, the results support that combination favors an acceptable safety profile and minimal additional toxicity compared with either single- vaccines or ICIs [18,19]. Emerging research is showing that it is possible to use new ICI molecules that function as vaccine molecular adjuvants. Here, we review the most recent advances in the use of ICIs-vaccines combinations, including mAbs and new strategies based on antisense oligonucleotides (ASOs), small non-coding RNAs, aptamers, peptides, and different small molecules for improving vaccine efficacy. It should be noted that the ordinary concept of vaccine adjuvant is a substance that is added to a vaccine to improve the magnitude and durability of the immune response. In the context of this review, several ICIs are included that are not part of the vaccine formulation, but when administered systemically they exert an adjuvant function. New approaches that include molecular ICIs as part of the vaccine formulation will also be addressed. In addition, although the main advances in the use of ICIs have been obtained in therapeutic antitumor vaccines, different research that is being carried out to evaluate the effect of certain ICIs in anti-infective vaccines will also be analyzed.

## 2. Overview of Immune Checkpoints

The immune response is the result of a delicate balance between several biological processes to protect the individual from pathogenic agents providing a suitable and optimal response. To achieve this balance, the immune system has developed several immune checkpoint pathways that comprise a wide range of molecules [20,21]. The overexpression of inhibitory receptors on cancer cells leads to a state of anergy and to a diminished immune response; therefore, blocking these molecules as a type of immunotherapy has been shown to improve the immune response against cancer. [22].

Immune checkpoints are often classified into first and second generations. The first generation include both CTLA-4 (CD152) and PD-1/PD-L1, while the second generation comprise the following molecules: lymphocyte activation gene-3 (LAG-3, CD223) [23], T cell immunoglobulin and mucin-domain containing-3 (TIM-3, CD366) [24], T cell immunoglobulin and ITIM domain (TIGIT) (CD112R) [25], V-domain Ig suppressor of T cell activation (VISTA) [26], B7 homolog 3 protein (B7-H3, CD276) [27], B and T cell lymphocyte attenuator (BTLA, CD272) [28], sialic acid-binding immunoglobulin-like lectin 15 (Siglec-15) [29], and CD39/CD73/adenosine pathway [30,31,32].

CTLA-4 is a transmembrane protein expressed on the surface of immune cells, such as T and B lymphocytes, natural killer (NK) cells, and NKT-like cells. This member of the CD28 family binds to CD80/CD86 on dendritic cells (DCs), memory B lymphocytes, and macrophages, inducing an inhibitory signal decreasing T lymphocytes activation, maturation, and proliferation through the phosphorylation of LAT and SLP76, with the consequent reduction in IL-2 production [33]. Another member of the CD28 family is PD-1 (CD279), a T cell (including NKT-like), NK, and B cell surface receptor. PD-1 has two ligands: PD-L1 (B7-H1 or CD274) and PD-L2 (B7-DC or CD273), which are found on APC, endothelial cells, fibroblasts, and bone marrow-derived mast cells and it can also be expressed by tumor cells [34,35,36]. After activation, the PD-1/PD-L1 pathway induces cell exhaustion and tolerance [35]. Another inhibitory receptor from the CD28 family that is gaining attention is TIGIT, which is expressed in NK cells, regulatory T cells (Tregs), and memory T cells [25,37]. TIGIT binds to two ligands: PVRL2 (CD112) and, with higher affinity, PVR (Necl-5, CD155). PVRL2 can be found in APCs and non-hematopoietic cells [38], while PVR has been found in DCs, fibroblasts, and endothelial cells [25]. Interestingly, these receptors are over-expressed in several types of cancers, inducing T cell suppression by interacting through the axis TIGIT/PVR in DCs [25,38]. In NK cells, it has been shown that TIGIT blockade prevents NK cell exhaustion and induces potent anti-tumor immunity, suggesting that its targeting might have anti-cancer therapeutic potential [39]. It is important to highlight that there is a binding competition for PVR (CD155) between TIGIT, and some stimulatory molecules such as DNAM-1 (CD226) and CD96 [40]. Another inhibitory molecule not belonging to the CD28 family is LAG-3. This receptor is expressed on T cells, NK cells, and B cells’ surfaces [41]. LAG-3 shares nearly 20% of structural motifs with CD4 and binds as well to the major histocompatibility complex class II (MHC-II) molecules on APCs, such as HLA-DR, HLA-DQ, HLA-DO, HLA-DP, and HLA-DM. However, LAG-3 has a 100-fold higher affinity for MHC-II than CD4 [41,42], thus resulting in a negative impact on the function of CD4 T cells [43]. In addition, LAG-3 regulates negatively CD4+T cells interfering in the TCR signaling pathway [44,45]. TIM-3 (CD366) is a surface receptor expressed on DC, monocytes, macrophages, mast cells, NK, and T cells. TIM-3 binds to galectin 9, phosphatidyl serine, HMGB1, or CEACAM-1, which are molecules produced by several tissue cells [40]. Several studies described the association of high expression of TIM-3 in cancer cells with a poor prognosis. Specifically, TIM-3 suppressed the anti-tumor function of CD8+ and CD4+ T cells [46,47,48] and inhibited the immune response mediated by Th1 cells [49]. In addition, TIM-3 induces peripheral immune tolerance [50,51] and its expression is a sign of exhaustion and apoptosis in T cells during chronic infections [52]. In contrast, in-vitro experiments showed that TIM-3 could enhance the immune response by inducing the transcriptional activity of NFAT/AP-1 and nuclear factor-kappa B (NF-κB) on T cell lines which increased IFN-γ production [53].

VISTA, also identified as PD-1 homolog (PD-1H), differentiation of embryonic stem cells 1 (Dies1), DD1α, Gi24, SISP1, B7-H5, and C10orf54, is a member of the transmembrane B7 family, with a single IgV domain with three extra cysteine residues (Cys44, Cys83, and Cys144). The main T cells inhibiting the function of VISTA reside on the surface-exposed histidine cluster. These specific features differentiate VISTA from other members of the B7 family with similar characteristics to PD-L1 and PD-L2 [26,54]. In the human immune system, the highest expression level of VISTA is detected in neutrophils, CD14+CD16+/− inflammatory monocytes, and CD11c+CD123^low^HLA-DR+ DCs. Moreover, in the lymphoid tissues, the main expression of VISTA is found in naïve CD4+ T cells, Tregs, and plasma cells. Several mAbs that can block the suppressive effects of VISTA are in commercial and therapeutic development [55].

B7-H3 is another immune checkpoint that is a member of the B7 family of immune checkpoint proteins [21]. B7-H3 is expressed on the surface of APCs, NK cells, and in a variety of tumor types. Besides its immunoregulatory role, B7-H3 has intrinsic pro-tumorigenic effects, such as promoting migration and invasion (metastasis), angiogenesis, chemoresistance, endothelial-to-mesenchymal transition, and tumor metabolism. Thus, overexpression of B7-H3 in tumor cells is related to poor prognosis. [56]. Several antibody-based strategies to target B7-H3-expressing cancer cells have been developed to achieve enhanced antitumor activity with acceptable safety profiles [57]. Finally, BTLA, Siglec-15, and CD39/CD73/adenosine pathways are new immune checkpoints that are being studied to promote the anti-tumor immune response [28,29,30,31,32].

## 3. Pharmacological Inhibition of Immune Checkpoints

To date, eight mAbs-based ICIs targeting PD-1 (nivolumab, pembrolizumab, cemiplimab, and dostarlimab), PD-L1 (atezolizumab, avelumab, durvalumab), and CTLA-4 (ipilimumab) have been approved in the European Union or the United States. Other products are currently under review or are being applied in specific countries, including China and Russia (Table 1). Despite their wide clinical use for cancer treatment, mAbs have some drawbacks that hinder their use and expansion, such as no oral bioavailability and poor permeation into cells due to their high molecular weights. Furthermore, mAbs therapies are related to Immune-Related Adverse Events (irAEs) [58] and the development of acquired resistance in some patients after prolonged treatments [59]. However, it has been demonstrated that increasing the number of activated infiltrating tumor-specific T cells by cancer vaccines can improve the success of ICIs therapy. Thereby, it has been proposed and validated that, a combination treatment of ICIs and cancer vaccines can lead to improving the antitumor immune responses [16,19,60].

**Figure 1:**
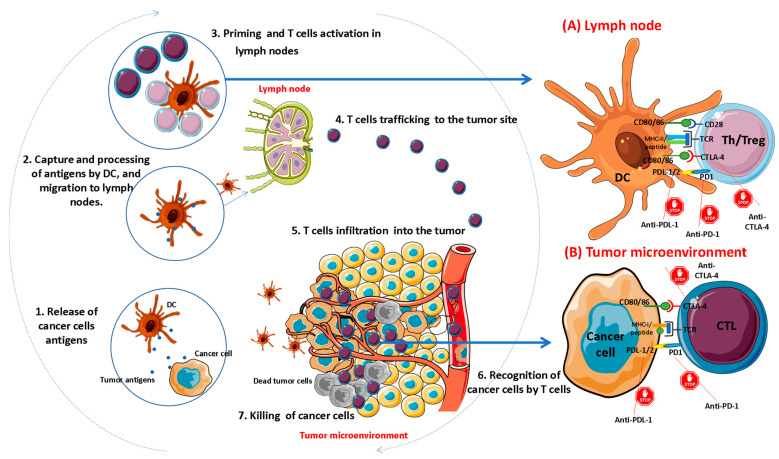
The Cancer-Immunity cycle is a process summarized in seven steps, which is initiated by the release of antigens from cancer cells and the activation of various immune mechanisms that ends with the elimination of cancer cells. The existence of negative feedback mechanisms developed both during the regulatory phase of the immune response (**A**), and by tumors to escape from the immune control (**B**), hinders this cycle and can be a hurdle to the development of effective immunotherapies. The goal of ICIs is to block critical immunosuppressive regulatory mechanisms and enhance effector T cells for the maintenance of the cycle of immunity against cancer. CTLs: Cytotoxic T lymphocytes; DC: Dendritic cell; MHC-I/MHC-II: Major Histocompatibility Complex Class I or II; PD-1: Programmed Death 1; PD-L1/2: Programmed Death-ligand either 1 or 2; Th: Helper T Cells; Treg: Regulatory T cell.

Most ICI-vaccine combinations have been used in the context of cancer vaccines, though new applications for anti-infectious vaccines are being investigated. Despite the limited efficacy of vaccine monotherapy, evidence shows that combination ICI-anti tumor vaccines can generate stronger tumor-specific immune responses associated with improved survival [16,19]. The rationale for combination immunotherapy lies in the “Cancer-Immunity Cycle”, which includes crucial points during anti-tumor response and consists of seven steps [20] (Figure 1). In the first step, the transformation of normal cells to cancer cells (oncogenesis) is associated with the release of neoantigens by dying tumor cells. These neoantigens include calreticulin (CRT), high mobility group box 1 (HMGB1), heat shock proteins (HSPs), ATP, and other structures. The released neoantigens are captured by DCs, via signals such as TLR4, CD92, and P2RX7. DCs maturate and migrate to draining lymph nodes (Step 2), process the protein antigens, and present them to prime and activate effector T cells (step 3). The activated T cells migrate to (Step 4) and infiltrate the tumor (Step 5). Within the tumor, the effector T cells recognize cancer cells (Step 6) and subsequently kill them (Step 7). Dying tumor cells continue to release more neoantigens, thus generating new cycles. In cancer patients, several factors may affect T cell priming and activation, including defective expression of MHC molecules in tumor cells, over-expression of inhibitory signals (CTLA-4, PD-1/PD-L1, TIM-3/phospholipids, BTLA, LAG3, IDO, Arginase), and stimulation of suppressive cells, such as Tregs, myeloid-derived suppressor cells, and M2 macrophages. Given the complicated immunity network in the tumor microenvironment and locoregional lymph nodes with the increased expression of immune checkpoints, it is reasonable to combine ICIs with tumor vaccines to promote the Cancer-Immunity Cycle activation. The use of ICIs can help overcome immune suppression in steps 3 and 6.

## 4. Combining ICIs and Anti-Tumor Vaccines

Numerous reports show that combined therapy with ICIs and cancer vaccines can promote increased immunogenicity and circumvent immunosuppressive activity in the tumor microenvironment. As occur with ICI immunotherapy, monoclonal antibodies against PD-1/PD-L1 and CTL-4 are the most widely used in the ICI-vaccine combination treatment [61]. Table 2 lists some selected studies where the combination of mAb-based-ICIs with antitumor vaccines were evaluated.

## 5. ICIs and Anti-Infectious Vaccines

Regarding anti-infective vaccines, there are not abundant reports of clinical investigations on the use of ICI to improve the efficacy of these vaccines compared to anti-tumor vaccines. However, there are several interesting reports of the effect of ICIs in neoplastic patients who have received anti-infectious vaccines. For example, ICI-vaccine combination has been studied in neoplastic patients who receive ICI therapy and are vaccinated with influenza, anti-COVID-19, and others anti-infectious vaccines. In 2018, Laubli et al. evaluated the serological responses to vaccination against Influenza A/H1N1, Influenza A/H3N2, and Influenza B/Brisbane at different time points after vaccine administration in cancer patients undergoing PD-1 blockade (either Nivolumab or Pembrolizumab) and healthy age-matched controls. The authors found that some patients treated with PD-1 blocking agents showed a rapid and significant increase of antibody titers. The seroconversion factors (the ratio between post- and pre-vaccine titers at day 30) were significantly higher in the cancer patients compared with the healthy controls. For influenza A/H1N1 antigen, the median seroconversion factor was 32 in cancer patients vs. 4 in healthy controls, (*p* = 0.02). For A/H3N2 antigen, the difference in the median seroconversion factor was also significant with 16 in cancer patients vs. 4 in healthy controls, (*p* = 0.03). In the case of the B/Brisbane antigen, the seroconversion factor was not statistically significant (*p* = 0.48) [83].

Similarly, a preclinical study in rhesus macaques showed a high frequency of specific T cells upon simian immunodeficiency virus (SIV) vaccination after PD-1 blockade [84]. A report at the American Society of Clinical Oncology meeting showed a significant increase in the specific IgM response after influenza vaccination in patients receiving anti-PD-1 treatment [85]. The authors studied the humoral response to a quadrivalent inactivated influenza vaccine in 28 patients treated with anti-PD-1 therapy. At 45 days, IgM responses to both influenza A and B common antigens were statistically significant (*p* < 0.05), while IgG response to common influenza B antigens was increased at day 45 (*p* = 0.001). There were no reported influenza-related hospitalizations, although one of 28 patients contracted influenza B infection. This suggests possible evidence of seroprotection.

In another study, Weber et al. evaluated the immunocompetence of patients with melanoma and receiving ipilimumab treatment [86]. The vaccine-specific immune response was evaluated after the administration of tetanus, influenza, and pneumococcal vaccines. The tetanus vaccine was administered 10 days before the beginning of ipilimumab, whereas influenza and pneumococcal vaccines were administered 5 days after the treatment. A significant increase in the percent of activated (HLA-DR+) CD4+ and CD8+ T cells with concomitant decreases in naive CD4+ and CD8+ T cells were observed in peripheral T-cell populations after ipilimumab treatment. These changes were evident by week 4 of treatment. The increases were also observed in central memory, effector memory, and activated ICOS+ CD4+ T cells, but not in ICOS+ CD8+ T cells or in FoxP3+ CD4 Tregs. However, ipilimumab treatment (3 or 10 mg/kg) had no impact on vaccine-induced humoral response.

Several recent systematic reviews have been carried out trying to find out the effect of ICIs on the efficacy and toxicity of vaccination against influenza in neoplastic patients [87,88,89,90]. In general, these studies showed that patients under ICI treatment who received influenza, pneumococcal, and anti-COVID 19 vaccines developed an apparently better humoral response with a higher rate of seroconversion [87]. However, vaccination appears to increase the risk of irAEs in patients undergoing ICIs treatment although no severe vaccination-related toxicities were reported.

Few studies have evaluated the effect of ICI during anti-COVID 19 vaccinations. Recently, Niewolik and collaborators observed an enhanced humoral immune response in patients under active or past ICI therapy after COVID-19 vaccination. They also observed a tendency for higher antibody levels when ICI therapy was received within the last six months before vaccination. Moreover, subgroup analysis revealed that patients under ongoing targeted therapy during the vaccination period had significantly higher median antibody levels than patients without any active antitumor treatment [91].

These recent reports open the perspective that the use of certain ICIs could serve as vaccine adjuvants to improve the immunogenicity and efficacy of certain anti-infective vaccines. In the following sections, we will discuss several studies that have shown this potential effect in laboratory animals.

## 6. Newly Emerging ICIs (Second Generation) for Combination Therapy

Although some ICIs have manifested convincing clinical effectiveness in certain tumor types, a large number of patients develop de novo or adaptive resistance. Aside from CTLA-4 and the PD-1/PD-L1, novel checkpoints have been discovered, which can be targeted by specific mAb in monotherapy and combination therapy. Numerous clinical trials of novel ICIs in cancer immunotherapy are ongoing or have been completed and they can be revised in reference [92].

In another recent report, Zahm et al. 2021, used an OVA-expressing mouse tumor model, to show that CD8 + T cells activated in the presence of APCs expressed multiple checkpoint receptors; but T cells activated without APCs expressed LAG-3 alone, suggesting that LAG-3 might be a preferred target in combination with vaccination. They also assessed the effects of peptide vaccines or DNA vaccines targeting three tumor antigens combined with blockade of PD-1 and/or LAG-3 on tumor growth in three different murine tumor models. In each model, the anti-tumor efficacy of vaccination was increased with PD-1 and/or LAG-3 blockade. However, vaccination combined with dual PD-1 and LAG-3 blockade induced the highest anti-tumor effect in a prostate cancer model in which PD-1 blockade alone with vaccination showed less efficacy [93].

In addition to the reports of the use of these second-generation ICIs in antitumor vaccines, Roy et al. demonstrated that exhausted herpes simplex virus type 1 (HSV-1)-specific CD8+ T cells, with high expression of LAG-3, were elevated in symptomatic patients with episodes of recurrent corneal herpetic disease. In this way, a therapeutic blockade of LAG-3 with antagonist antibodies combined with a therapeutic immunization with gB498-505 peptide, the immunodominant epitope of HSV-1, in latently infected mice, significantly restored the quality and quantity of functional gB498-505 specific CD8+ T cells in both trigeminal ganglia and cornea and protected against UV-B induced recurrent corneal herpes infection and disease [94].

## 7. New Alternatives of Immune Checkpoint Inhibition

Despite the widespread use of monoclonal antibodies as the main route for blocking the immune checkpoint, these compounds have several drawbacks. For this reason, low-molecular-weight inhibitors are a promising and highly demanded alternative for the mAbs therapeutics, which could serve as inexpensive tools to overcome these disadvantages. A comparison between mAbs and alternative molecular ICIs are shown in Table 3. Next, we will review some of the most promising emerging molecular ICIs.

### 7.1. ASOs

ASOs are short, synthetic, single-stranded sequences of deoxyribonucleotides that can target any gene product of interest. Typically, an ASO is an oligonucleotide with a mean length of 12 to 25 nucleotides, complementary to the sequence of the target gene’s transcribed messenger RNA (mRNA). These molecules can be localized in both the nucleus and cytoplasm, making it possible to reach their targets in different cellular localization [95,96]. The cell has nucleases capable of degrading nucleotide chains; therefore, ASOs are chemically modified to protect them against the action of these enzymes. The chemical modifications of the ASOs are important because, in addition to protecting against enzymatic degradation, they determine their trafficking and mode of action [97]. The chemical modification also establishes the evolution of ASOs: phosphorothioate linkages (first generation); sugar modifications, including 2′-O-methyl (2′OMe) and 2′-methoxyethyl (MOE) modifications (second generation); locked nucleic acid [LNA]; and morpholino phosphorodiamidate [MF], phosphorodiamidate morpholino oligomer [PMO], and peptide nucleic acid [PNA] [98]. Once inside the cell, the ASO binds to the target mRNA or pre-mRNA, inducing its degradation and preventing the translation of a specific protein. Therefore, ASOs offer huge opportunities for pharmacological modulation by controlling the production of specific proteins revolutionizing personalized medicine. Several ASOs have received marketing authorization from the drug agencies (Food and Drug Administration (FDA) in the United States of America and the European Medicines Agency (EMA) in Europe) to treat diverse diseases caused by genetic alterations [99].

The use of ASOs as vaccine adjuvants is a newly emerging area of research [100]. ASOs as vaccine adjuvants have been designed against diverse types of suppressor components such as transcription factors [101,102], cytokines [103,104,105], and immune checkpoints [101,106,107].

In 2017, Miguel et al. evaluated whether combining therapeutic vaccination with either CTLA-4 or Foxp3 ASOs can improve the antitumor response in C57BL6 mice. The mice were immunized with irradiated B16 tumor cells engineered to produce granulocyte-macrophage colony-stimulating factor (GM-CSF). Different groups of mice were intraperitoneally treated with either CTLA-4 or Foxp3 ASOs before and after vaccination. Mice survival, tumor growth, and CTLA-4/Foxp3 expression in peripheral blood cells were measured. An improved survival effect was achieved by combining the therapeutic vaccine with Foxp3 ASOs or CTLA-4 ASOs (50% and 20%, respectively). Interestingly, both peripheral blood CD4+CD25+Foxp3+ and CD4+CTLA-4+ cell counts were higher in mice that developed tumors on the day of sacrifice [101].

In that same year, 2017, two ASOs against CTLA-4 (named CMD-1 and CMD-2), were analyzed to evaluate their synergistic effect in experimental vaccines prepared with either inactivated foot-and-mouth disease virus (FMDV) or recombinant porcine circovirus type 2b (PCV2b) capsid protein in mice. The sequences of both CMD-1 and CMD-2, were complementary to 3′ untranslated region (3′ UTR) conserved regions that are identical between mouse and human CTLA-4 mRNA [106]. CMD-1 down-regulated the antigen-induced CTLA-4 activation on CD4+ T cells and enhanced the antibody response against both PCV2b and FMDV vaccines in Balb/c and ICR mice compared with the control group without ASOs (*p* < 0.05). Moreover, CMD-1 triggered a high recalled proliferation of CD4+ T lymphocytes and CD19+ B lymphocytes and elevated expression of CD80 and CD86 on the CD11c+ populations.

Two years later, the same group reported the effect of an interfering ASO (LIO-1) against LAG-3 to enhance the immune response induced by two types of influenza vaccines [107]. The authors demonstrated that LIO-1 induced the degradation of LAG-3 mRNA and decreased the LAG-3 expression on CD4+ T cells. LAG-3 blocking promoted the activation production of IFN-γ, IL-2, and IL-6 by CD4+ T cells re-stimulated with specific antigens. Furthermore, LIO-1 enhanced the antibody responses induced by both vaccine formulations in mice.

### 7.2. Small Non-Coding RNAs

Small non-coding RNAs are a type of non-coding transcripts that participate in regulating gene expression at the transcriptional and/or posttranscriptional levels. So far, three types of small non-coding RNAs have been described: micro-RNA (miRNA), small interfering RNA (siRNA), and Piwiinteracting RNA (piRNA). Other non-canonical small non-coding RNAs, tRNA-derived small RNA (tsRNA) and rRNA-derived small RNA (rsRNA), have been much less investigated [108]. siRNA, sometimes named short interfering RNA or silencing RNA, is a class of double-stranded non-coding RNA molecules, of 20–24 base pairs in length. siRNA interferes with the expression of specific genes with complementary nucleotide sequences by targeting and degrading complementary mRNA transcripts and defending against foreign nucleic acids. Therefore, they contribute to the posttranscriptional regulation of gene expression [109].

In 2013, Hobo et al. evaluated the generation of a DC vaccine with improved immunogenic potential by combining PD-1 ligand siRNA. They demonstrated that PD-L1/PD-L2 siRNA delivery using DLin-KC2-DMA-containing lipid nanoparticles (LNP) mediated an efficient PD-L1/2 knockdown on human monocyte-derived DC. The transfection method did not affect DC viability, phenotype, or migratory capacity. Moreover, they demonstrated that PDL-silenced DC loaded with antigen mRNA superiorly boosts ex vivo antigen-specific CD8(+) T cell responses from transplanted cancer patients [110]. In another report, Roeven et al. developed a highly effective clinical-grade DC vaccine by combining PD-L siRNA delivery through SAINT-RED with minor histocompatibility antigens (MiHA)-encoding mRNA pulsing. SAINT-RED is a known delivery reagent for nucleotides and proteins that contain the cationic amphiphilic lipid SAINT-18 (1-methyl-4-(cis-9-dioleyl) methyl-pyridinium-chloride) and the neutral helper-lipid dioleoylphosphatidylethanolamine (DOPE) in a 1:1 molar ratio [111]. The authors showed that the transfection method might be combined with target antigen mRNA or peptide loading to efficiently stimulate MiHA-specific T-lymphocytes without affecting DCs viability or phenotype. These MiHA mRNA pulsed PDL-silenced DC exhibited superior stimulatory potential to boost MiHA-specific T-cell proliferation and cytokine production. They also reported that DCs vaccination expanded adoptively transferred antigen specific CD8(+) T cells in vivo. In addition, PD-L silenced DCs improved boosting and further expansion of ex vivo primed MiHA-specific CD8(+) T lymphocytes in immunodeficient mice [112]. In 2017, Jadidi-Niaragh et al. evaluated the efficacy of CD73-specific siRNA-loaded chitosan-lactate nanoparticles in combination with tumor lysate-pulsed DCs vaccine for the treatment of 4T1 breast cancer-bearing mice. Intravenous administration of CD73-specific siRNA-loaded NPs led to reduced expression of CD73 in tumor cells which was associated with decreased tumor growth and metastasis, and increased mice survival [113]. They also observed downregulation of Tregs, myeloid-derived suppressor cells (MDSCs), and tumor-associated macrophages with reduced levels of IL-10. These changes were associated with an augmented CTL effector function, improved proliferative response of T cells, and increased production of IFN-γ and IL-17. Moreover, this treatment attenuated the expression and activities of matrix metalloproteinases (MMPs) 2 and 9 associated with the prevention of lung metastasis.

Wang et al. developed an experimental cancer vaccine that simultaneously introduced an mRNA encoding a melanoma-associated antigen, tyrosinase-related protein 2 (TRP2), and a PDL-1 blocking siRNA into the APCs. They used a lipid-coated calcium phosphate (LCP) nanoparticle (NP) as a carrier. The lipid NPs were formulated with mannose to facilitate the preferential uptake by DCs in the lymph nodes after subcutaneous administration. The simultaneous delivery of the mRNA vaccine with PD-L1 siRNA downregulated PD-L1 in the DCs that presented tumor antigens. The vaccine significantly promoted T lymphocytes activation and elicited a vigorous antigen-specific cytotoxic T lymphocytes response and a humoral immune response in a mouse model of B16F10 melanoma. The immune responses inhibited melanoma growth. The enhanced T cell response had a profound inhibitory effect on tumor growth and metastasis [114]. More recently, Esmaily et al. used a siRNA-loaded chitosan-lactate nanoparticles to suppress the expression of CTLA-4 on tumor-infiltrating T lymphocytes. These nanoparticles were prepared to facilitate priming anti-tumor T lymphocytes induced by a tumor lysate-loaded DC vaccine. The administration of anti-CTLA-4 siRNA-loaded NPs into CT26 and 4T1 tumor-bearing mice led to the downregulation of CTLA-4 on tumor-infiltrating T lymphocytes. It was associated with regression of the tumor and increased survival of tumor-bearing mice compared with the group treated with DC vaccine alone. Thus, the authors showed that the silencing of CTLA-4 can potentiate the T lymphocyte priming capacity of the DC vaccine [115]. In another study, the same group silenced the expression of PD-L1 in DCs and PD-1 in T lymphocytes, once again using the siRNA-loaded NPs platform and evaluated the DC phenotypic and functional characteristics and T-c lymphocyte functions following tumor antigen recognition on DCs, ex vivo. They showed that synthesized NPs were efficiently uptake by cells and induced target gene silencing. Presentation of tumor antigens by PD-L1-negative DCs to PD-1-silenced T lymphocytes led to the induction of potent cellular responses [116]. Several additional reports have confirmed that the use of siRNA silencing immune checkpoints are a promising tool for a new generation of therapeutic cancer vaccines [117,118,119].

### 7.3. Aptamers

Aptamers are small (usually from 20 to 60 nucleotides) single-stranded RNA or DNA oligonucleotides that bind their targets with high specificity and affinity due to their three-dimensional structures. Aptamers are essentially a chemical equivalent of antibodies, but they have the advantage of being relatively smaller in size, and non-immunogenic [120].

In 2003, the group led by Eli Gilboa reported the first demonstration of using aptamers to manipulate the immune system in vivo. Using affinity-based in vitro selection methods, they isolated short aptamers that bind murine CTLA-4 with high affinity and specificity, blocking its function in vitro and in vivo. However, compared with the anti-CTLA-4 antibody, more aptamer was required to elicit inhibition of tumor growth in vivo. In their pioneer studies, they noticed that although the avidity of the aptamers to their CTLA-4 targets is comparable if not superior to that of the anti-CTLA-4 antibody, the bioavailability of the aptamers is significantly lower in vivo, being necessary to administer higher doses (10–15 nmol/mouse) [121]. The subsequent efforts were directed to the search for delivery systems that would allow optimizing the biodistribution of these molecules [122]. In this way, Prodeus et al. developed a DNA aptamer (MP7) that binds specifically to the extracellular domain of PD-1 and blocks the PD-1–PD-L1 interaction. MP7 functionally inhibited PD-L1-mediated suppression of IL-2 secretion in primary T lymphocytes, while a PEGylated form of MP7 retained the ability to block the PD-1–PD-L1 interaction, and significantly suppressed the growth of PD-L1+ colon carcinoma cells in vivo with a potency equivalent to an anti-PD-1 antibody. No off-target TLR-9-related immune responses were observed associated with anti-PD-1 DNA aptamer treatment [123]. After that, Lai and collaborators reported that a DNA aptamer against human PD-L1 blocked the binding between PD-1 and PD-L1, promoted lymphocyte proliferation in vitro, and suppressed tumor growth in vivo with minimum renal and liver toxicity. Tumors treated with the aptamer showed increased infiltration of CD4+ and CD8+ T cells and higher levels of IL-2, IFNγ, TNFα, and C-X-C chemokine ligands, such as CXCL9 and CXCL10 and CXCR3 expression in the CD8+ T cells than control [124]. Similar results were recently shown by Gao et al. using a model of CT26 colon carcinoma [125].

Other aptamers have been developed against TIM-3 in combination with PD-L1 inhibitor with a synergistic effect in colon carcinoma-bearing mice [126,127], and against LAG-3 [128]. However, although there are several reports of the use of aptamers in cancer immunotherapy, there are still not enough reports of their use in combination with vaccines. Given the demonstrated effects of aptamers as modulators of the immune response, it is anticipated that there will be advances in this area in the future.

### 7.4. Peptides and Other Small-Molecule ICIs

Within the last few years, significant advances have been achieved on the development of peptides and small-molecule ICIs, with special focus in the PD-1/PD-L1 axis. Currently, diverse small-molecule inhibitors based on three different therapeutic approaches interfering PD-1/PD-L1 signaling pathway are being investigated. These inhibitors can act by: (1) blocking direct interaction between PD-1 and PD-L1; (2) inhibiting transcription and translation of PD-L1; and (3) promoting degradation of PD-L1 protein [129]. It is beyond the scope of this review to address the numerous small-molecule inhibitors that are being studied. For more details, we refer readers to several recent reviews that have summarized this progress [130,131,132,133,134,135,136,137]. Another interesting approach is the use of SCH58261, a new generation of A2A adenosine receptor ICI blocker that inhibits the immunosuppressive adenosinergic pathway in the tumor microenvironment, activating NK cells and CD8+ T cells, and inhibiting the proliferation of Tregs. In 2017, Arab et al. reported an increased efficacy of a dendritic cell-based therapeutic cancer vaccine combined with SCH58261 and the CD73 inhibitor adenosine 5-(α,β-methylene) diphosphate in a mouse breast tumor model. They demonstrated that inhibition of CD73 activity, both individually and, especially, in combination SCH58261, enhanced the potency of DC vaccines to induce more effective antitumor immune responses, inhibit retarded tumor growth, and prolong the survival of tumor-bearing mice [138]. More recently, it was revealed that SCH58261 combined with a nanovaccine based on redox-responsive polymer micelles lead to 47.6% down-regulation of the level of CD4+Foxp3+ Tregs and 85.0% decrease of the content of TGF-β, and induced a sixfold increase of the number of CD8+ T cells and twofold up-regulation of NK1.1+CD49b+ NK cells. This formulation displayed an appropriate immunotherapy efficacy with a tumor growth inhibition rate (TGI) of ≈89.9% and even a complete regress of 40% of tumors [139].

## 8. Future Directions and Closing Remarks

The design and engineering of different types of ICIs as molecular adjuvants for personalized vaccines have gained significant momentum in recent years, due to the accumulation of predictable and therapeutically promising molecular targets. The vast information that exists on the benefits of using ICIs to improve the efficacy of vaccines has focused on mAbs, and especially on tumor vaccines. Despite their wide use, mAbs present multiple problems to be used as vaccine adjuvants, for this reason, the use of smaller molecules capable of acting not only on the cell surface, but also inside the cell and with adequate biodistribution and a low toxicity profile has been a high priority in recent years. Among the molecules that have been most studied are ASOs, siRNA, aptamers, peptides, and other small-molecule ICIs. So far, these technologies have progressed rapidly from an academic discovery to a potential new class of treatment for human disease. Increasing studies reveal that the inhibition of immune checkpoints such as CTLA-4, PD1/PDL1, and other new emerging pathways induce a better response to the vaccine, although in some cases this may be associated with some types of irAEs. Off-target effects and other toxicity issues of new molecules are major hurdles that need to be addressed as knowledge in this area advances. Delivery of these new molecules, especially when systemically administered, is another important barrier to be overcome. New materials and carrier systems are being investigated to enhance delivery efficiency, but approval procedures could be hindered by the standardization of complicated formulations. However, the rapid advances that are being made in drug design, taking advantage of the use of bioinformatic methods associated with high-precision technologies, suggest that these obstacles will be resolved, and, in a few years, a new generation of vaccine adjuvants that exploit the regulatory pathways of the immune response will be used to obtain more effective and safer vaccines.

## Figures and Tables

**Table 1:** Therapeutic monoclonal antibodies-based ICIs approved or in regulatory review in the European Union (EU) or United States (US).

	Target; Format	1st Indication Approved/Reviewed	1st EU Approval Year	1st US Approval Year
Ipilimumab	CTLA-4; Human IgG1	Metastatic melanoma	2011	2011
Pembrolizumab	PD1; Humanized IgG4	Melanoma	2015	2014
Nivolumab	PD1; Human IgG4	Melanoma, non-small cell lung cancer	2015	2014
Atezolizumab	PD-L1; Humanized IgG1	Bladder cancer	2017	2016
Avelumab	PD-L1; Human IgG1	Merkel cell carcinoma	2017	2017
Durvalumab	PD-L1; Human IgG1	Bladder cancer	2018	2017
Cemiplimab	PD-1; Human IgG4	Cutaneous squamous cell carcinoma	2019	2018
Dostarlimab	PD-1; Humanized IgG4	Endometrial cancer	2021	2021
Relatlimab	LAG-3; Human IgG4	Melanoma	Review	2022
Tremelimumab	CTLA-4; Human IgG2A	Antineoplastic; liver cancer	Review	Review
Tislelizumab	PD-1; Humanized IgG4	Esophageal squamous cell carcinoma	Review	Review
Sintilimab	PD-1; Human IgG4	Non-small cell lung cancer	NA	Review
Retifanlimab	PD-1; Humanized IgG4	Squamous cell carcinoma of the anal canal	MAA withdrawn	Review
Penpulimab	PD-1; Humanized IgG1	Metastatic nasopharyngeal carcinoma	NA	Review
Omburtamab	B7-H3; Murine IgG1	CNS/leptomeningeal metastasis from neuroblastoma	Review	Review
**Other regions**				
Sintilimab (Tyvyt): human anti-PD-1 mAb approved in China in December 2018 for Hodgkin’s lymphoma; Toripalimab (Tuoyi): humanized anti-PD-1 mAb approved in China in December 2018 for melanoma; Camrelizumab: humanized anti-PD-1 mAb approved in China in 2019 for Hodgkin’s lymphoma; Tislelizumab: humanized anti-PD-1 mAb, approved in China in December 2019 as a treatment for classical Hodgkin’s lymphoma; Disitamab vedotin (Aidixi): anti-HER2 humanized ADC approved in China in June 2021 as a treatment for gastric cancer; Penpulimab: ant-PD-1 humanized mAb approved in China in August 2021 for Hodgkin’s lymphoma; Zimberelimab: anti-PD-1 human mAb approved in China in August 2021 for Hodgkin’s lymphoma; Prolgolimab (Forteca), anti-PD-1 mAb approved in Russia in 2020 for melanoma.				

Source: https://www.antibodysociety.org/resources/approved-antibodies/ (accessed on 8 August 2022).

**Table 2:** Selected studies with combination of vaccines and monoclonal antibodies- based ICIs.

ICI/Target	Vaccine	Disease	*n*	ICI-Vaccine Combination Strategy	Results	irAEs	References
Ipilimumab/CTLA-4	T-VEC	Unresectable stage IIIB-IV melanoma	198	T-VEC was administered intratumorally at the first dose ≤4 mL × 10^6^ pfu/mL, after 3 weeks at subsequent doses ≤4 mL × 10^8^ pfu/mL every 2 weeks; four doses of ipilimumab 3 mg/kg were given intravenously every 3 weeks.	Combination therapy promoted a significantly higher objective therapeutic response rate than ipilimumab alone (39% vs. 18%). A decrease in visceral lesions was observed in 52% of patients treated with the combination and 23% of patients with ipilimumab alone.	Fatigue (59%), chills (53%), diarrhea (42%), pruritus (40%), rash (39%). The incidence rate of grade ≥3 irAEs was 45% in the combination and 35% with ipilimumab alone.	[62,63]
	Sipuleucel-T	mCRPC	50	All patients received 3 doses of intravenous Sipuleucel-T infusion once every 2 weeks. Patients received the first dose of ipilimumab either immediately following their last Sipuleucel-T infusion, or 3 weeks after their last vaccine infusion. Additional 3 doses of ipilimumab, 3 mg/kg was given to all patients every 3 weeks, for a total of 4 ipilimumab doses	The combination treatment induced CD4(+) and CD8(+) T lymphocytes activation that was most pronounced with the immediate schedule. Lower frequencies of CTLA-4(+) circulating T lymphocytes, were associated with better clinical outcomes. However, combining Ipilimumab with Sipuleucel-T resulted in modest clinical activity.	The treatment was well tolerated. One patient underwent a grade 4 event (colitis with colonic perforation) and nine grade 3 events in seven patients. Interestingly, patients with an irAE were more likely to have a significant PSA response (any grade, *p* = 0.001, grade 3/4, *p* = 0.037).	[64]
	PROSTVAC	mCRPC	30	PROSTVAC was administered subcutaneously at prime doses of 2 × 10^8^ pfu/mL, with subsequent monthly doses of 1 × 10^9^ pfu/mL. Intravenous ipilimumab was given (1, 3, 5, and 10 mg/kg) on the same day as the vaccine.	For patients receiving ipilimumab 10 mg/kg, overall survival was 37.2 months, very longer than historical controls of treatment with PROSTVAC or ipilimumab alone.	irAEs mostly occurred in patients treated with ipilimumab 10 mg/kg. Grades 1 to 2 injection-site reactions were the most common adverse events. Grades 3 to 4 irAEs, including rash, diarrhea, colitis, and endocrine events, were observed in 27% of patients, requiring replacement hormones or supportive measures	[65,66]
	GVAX	mCRPC	28	All patients received GVAX intradermally. Priming dose of 5 × 10^8^ cells with additional injections of 3 × 10^8^ cells every 2 weeks for 24 weeks plus intravenous ipilimumab at doses of 0.3, 1, 3, and 5 mg/kg every 4 weeks.	25% of patients showed >50% PSA reduction from baseline, and four patients obtained stable disease measured by bone scan.	Adverse events (>30%) were grades 1 to 2 injection-site reactions, fatigue, influenza-like symptoms, and rash. At, one patient receiving ipilimumab at 5 mg/kg, had grade 4 sarcoid alveolitis. Other irAEs related to ipilimumab included hypophysitis and hepatitis. Both responded to hormone replacement therapy.	[67,68]
		Advanced pancreatic adenocarcinoma	30	Patients received either intravenous ipilimumab 10 mg/kg alone or intradermal GVAX at doses of 5 × 10^8^ cells with subsequent ipilimumab 10 mg/kg.	The combination promoted prolonged disease stabilization, improved 1-year survival (27% vs. 7%), and a trend of favorable median overall survival (5.7 vs. 3.6 months; *p* = 0.072) compared with ipilimumab alone. CA19-9 responses were observed in 47% of patients that received combination therapy, whereas none in ipilimumab alone.	Grades 1 to 2 injection-site reactions, rash, fatigue, fever, and influenza-like illness. 20% of patients experienced grades 3 to 4 irAEs including rash, colitis, pneumonitis, and nephritis. All irAEs responded to steroids except for nephritis requiring hemodialysis	[69]
	Peptide Vaccine (gp100:209-217 and gp100:280-288 from gp100, a melanoma-associated antigen.	Progressive stage IV melanoma	56	Twenty-nine patients received 3 mg/kg Ipilimumab every 3 weeks, whereas 27 received 3 mg/kg as their initial dose with subsequent doses reduced to 1 mg/kg every 3 weeks. The patients received concomitant vaccination with peptide vaccine.	Two patients had a complete response (at 30 and 31 months, respectively) while five patients achieved a partial response, for an overall objective response rate of 13%. Tumor regression was seen in lung, liver, brain, lymph nodes, and subcutaneous sites, and it was correlated with autoimmune reactions.	Of 14 patients with grade 3/4 autoimmune reactions, 36% experienced favorable clinical response. Only two favorable responses were observed in the 42 patients (5%) with no autoimmune reactions (*p* = 0.008). There were no significant differences in response rate or toxicity between the two-dose schedules.	[70]
	TriMixDC-MEL	Pretreated advanced melanoma	39	TriMixDC-MEL was given subcutaneously and intravenously plus ipilimumab (10 mg/kg) every 3 weeks for four doses, followed by nivolumab (anti-PD1) maintenance every 3 months	The disease control rate was 51% at 6 months, and tumor objective response rate with the combination was 38%, which was higher than ipilimumab alone (10–15%). Tumor responses included eight complete and seven partial responses.	The most common adverse events (>30%) were injection-site reactions, influenza-like illness, dermatitis, and chills. 14 patients (36%) underwent grades 3 to 4 events, but most of them were reversible by using established treatment.	[71]
	UV1	Unresectable metastatic melanoma	12	Ipilimumab (3 mg/kg) was administered every 3 weeks for a total of 4 doses. Intradermal abdominal injections of UV1 vaccines (300 µg doses) were administered as before and between treatments of ipilimumab. Thereafter every fourth week up to 28 weeks, and at weeks 36 and 48. GM-CSF (sargramostim 75 µg) was injected at the same site 10–15 min prior to UV1.	Ten patients showed a Th1 immune response to UV1, occurring early and after a few vaccinations. Three patients obtained a partial response. One patient had a complete response. Overall survival was 50% at 5 years.	The adverse events observed were injection site reaction, pruritus, rash, nausea, diarrhea, and fatigue.	[72]
Atezolizumab/PDL-1	Sipuleucel-T	mCRPC		Patients received either atezolizumab 1200 mg intravenously every 3 weeks for 2 doses followed by Sipuleucel-T three infusions every 2 weeks, or Sipuleucel-T every 2 weeks for a total of three infusions followed by atezolizumab as described (Phase Ib study)	The primary endpoint of this study was safety. There were no grade 5 adverse events attributed to the study drugs. Two patients underwent grade 4 toxicities, while eight grade 3 toxicities and four grade 3 toxicities were observed	None of the grade 3 or 4 adverse events were irAEs.	[73]
Pembrolizumab/PD1	T-VEC	Unresectable stages IIIB-IV melanoma	21	Patients received T-VEC at an initial dose of 4 mL × 106 pfu/mL, followed 3 weeks later at a full dose of 4 mL × 108 pfu/mL every two weeks. Pembrolizumab 200 mg was injected intravenously coinciding with subsequent doses of T-VEC.	Combination therapy induced an objective response rate of 62%, almost twice as shown in the phase III study of pembrolizumab (34%) and T-VEC (26%). The complete response rate for per immune-related response criteria was 33%. An increase in lymphocyte infiltration, and IFN-γ gene expression was observed in patients who responded to combination therapy.	The most common adverse events observed were. fatigue (62%), chills (48%), fever (43%), rash (33%), and arthralgia (33%). One grade 1 reaction associated with the combination resulted in hospitalization, while other grades 3 to 4 AEs were due to pembrolizumab. In general, combination therapy did not increase the toxicity of monotherapy.	[74,75]
		Advanced squamous cell carcinoma of the head and neck	36	T-VEC was injected intralesionally at a first dose of 8 mL × 10^6^ pfu/mL, and subsequent doses of 8 mL × 10^8^ pfu/mL every 3 weeks. Intravenous pembrolizumab 200 mg was administered every 3 weeks	The objective response rate was 16.7% (six patients with five subjects PDL-1 positive), and the disease control rate was 38.9% (14 patients with 11 subjects PD-L1 positive).	The detected adverse events included pyrexia (36.1%), dyspnea (33.3%), and fatigue (25.0%). Grades 3 to 4 reactions were observed in 24 patients (66.7%).	[76,77]
Nivolumab/PD1	Peptide Vaccine (MART-1/NY-ESO-1/gp100 with Montanide ISA 51 VG)	Unresectable stages III to IV melanoma	90	In a phase I trial, patients were treated with an extended dose of nivolumab (1, 3, or 10 mg/kg) with or without vaccines.	For both ipilimumab-refractory and -naive subjects, the RECIST (Response Evaluation Criteria in Solid Tumors) response rates were 25%, and nivolumab-induced durable responses for up to 140 weeks	Fatigue and injection-site reaction were the most common adverse events, most of which were mild to moderate. Grade 3 irAEs (optic neuritis, fever, pneumonitis, and rash) were also observed and were successfully treated with prednisone as described previously for nivolumab monotherapy.	[78,79]
		Resected stages IIIC to IV melanoma	33	Patients were treated with an extended dose of nivolumab (1, 3, or 10 mg/kg) plus peptide vaccine every 2 weeks for 24 weeks, followed by nivolumab alone every 3 months for up to 2 years	The estimated median relapse-free survival (RFS) was 47.1 months compared with the historical median RFS (12–21 months).	Injection-site reaction, fatigue, rash, pruritus, nausea, and arthralgia were the most common reactions registered (>40%). Grade 3 reactions included hypokalemia, rash, enteritis, and colitis. All of them responded to systemic management of steroids and supportive care	[80,81,82]

Legends: CA19-9: One of the most common tumour markers used in gastrointestinal diseases. It is the marker most used for pancreatic cancer; CTLA-4: Cytotoxic T-Lymphocyte Antigen 4; irAEs: Immune-Related Adverse Events; mCRPC: Metastatic Castration-Resistant Prostate Cancer; GVAX (Aduro BioTech): pancreatic cancer vaccine consisting of either autologous or allogeneic whole tumor cells, genetically modified to secrete GM-CSF, and then irradiated to prevent cell division; GM-CSF: granulocyte-macrophage colony-stimulating factor; pfu/mL: plaque forming units per milliliter; Prostvac (Bavarian Nordic): prostate cancer vaccine regimen consisting of recombinant poxviruses expressing PSA and costimulatory molecules (B7.1, ICAM-1, and LFA-3; PSA: prostate-specific antigen; Sipuleucel-T (Provenge^®^): An autologous therapeutic vaccine developed to treat prostate cancer; TriMixDC-MEL: Autologous monocyte-derived dendritic cells vaccine (DCs) electroporated with synthetic mRNA encoding CD40 ligand (CD40L), CD70 and a constitutively activated TLR4; T-VEC: Talimogene laherperepvec (T-VEC) is an injectable modified oncolytic herpes virus being developed for intratumoral injection; UV1: A synthetic cancer vaccine consists of three long peptides of the human telomerase reverse transcriptase catalytic subunit (hTERT).

**Table 3:** Comparison between monoclonal antibodies (mAbs) and alternative molecular ICIs (antisense oligonucleotides (ASOs), small non-coding RNAs, aptamers, peptides, and different small molecules).

	mAbs	Alternative Molecular ICIs
Specificity	Highly specific but cross-reactivity can be observed	Highly specific but off-target interaction can be observed
Diversity	mAbs can be obtained against a very wide range of target structures	
Mechanisms of action	Specific blockade of the checkpoint’s interaction with their natural ligand on the cell surface	Blocking direct interaction between checkpoint’s interaction with their natural ligand, inhibiting transcription and translation of checkpoint; promoting checkpoint degradation
Purity	High purity	High purity
Molecular weight	High molecular weight (~150 kDa)	Low molecular weight (~6 to10 kDa)
Structure	Glycoproteins with complex structure	Short, single-stranded DNA or RNA with chemical modifications, short linear peptides, cyclopeptides, or small synthetic molecules
Thermal Stability	Low stability. Cold chain through the storage, handling, and transportation is necessary	Highly stable. Lyophilization and freezing do not modify their biological activity
Bioavailability	No oral bioavailability and inability to penetrate the cells. The Fc domain of IgG antibody can interact with diverse cell receptors which hinder reaching the targets	They can penetrate the cells and act on intracellular targets
Secreted target	Secreted targets (e.g from tumor cells), can interrupt antibody-mediated immune reactions in the tumor microenvironment.	Their targets are mainly intracellular
Immunogenicity	Highly immunogenic by xenogeneic differences, e.g., between mice and humans	They are not properly immunogenic
Toxicity	Different grades of toxicity have been described	Relatively low toxicity associated with off-target effects
Development and Manufacturing	Immortal hybridomas cell lines produce unlimited quantities of antibodies, but industrial production is technologically complex	They are obtained synthetically. The use of vehicles can add complexity to the manufacturing process

## Data Availability

Not applicable.
